# Supplementary material for: A High-Quality Reference Genome Assembly of Prinsepia uniflora (Rosaceae)
Source: Genes (Basel). 2023 Nov 2;14(11):2035. doi: 10.3390/genes14112035 (PMC10671140; doi:10.3390/genes14112035)
Supplement: Supplementary file 1 [file genes-14-02035-s001.zip › genes-2660517-supplementary.pdf]

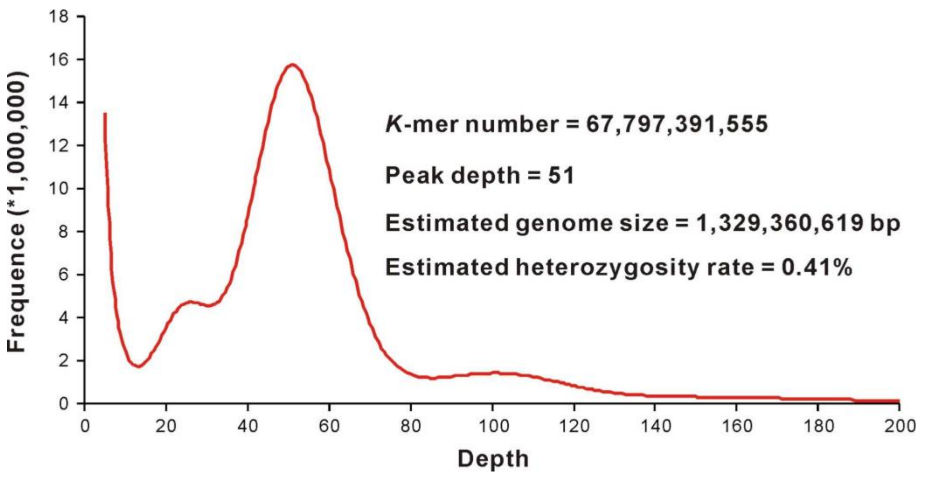

Figure S1. A 19-mer frequency distribution of *P. uniflora* genome based on Illumina paired-end reads.

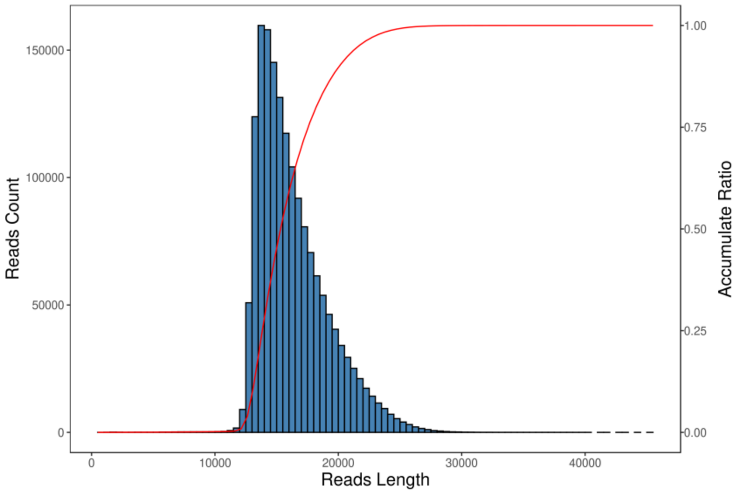

Figure S2. Subreads length distribution of PacBio HiFi data.

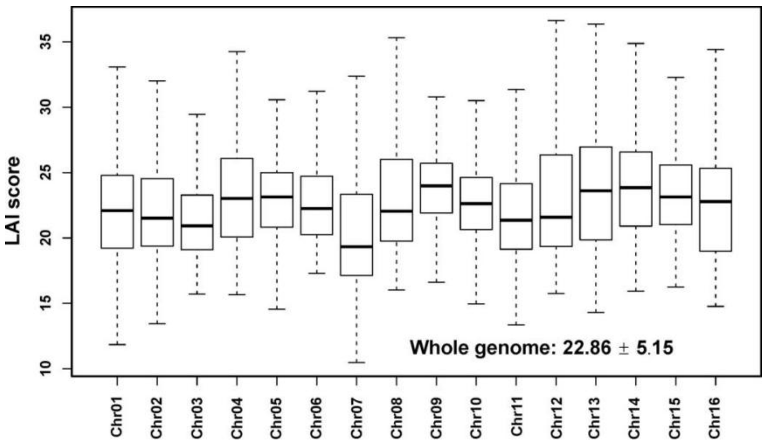

Figure S3. Distribution of LAI score in the 16 pseudochromosomes of *P. uniflora* genome.

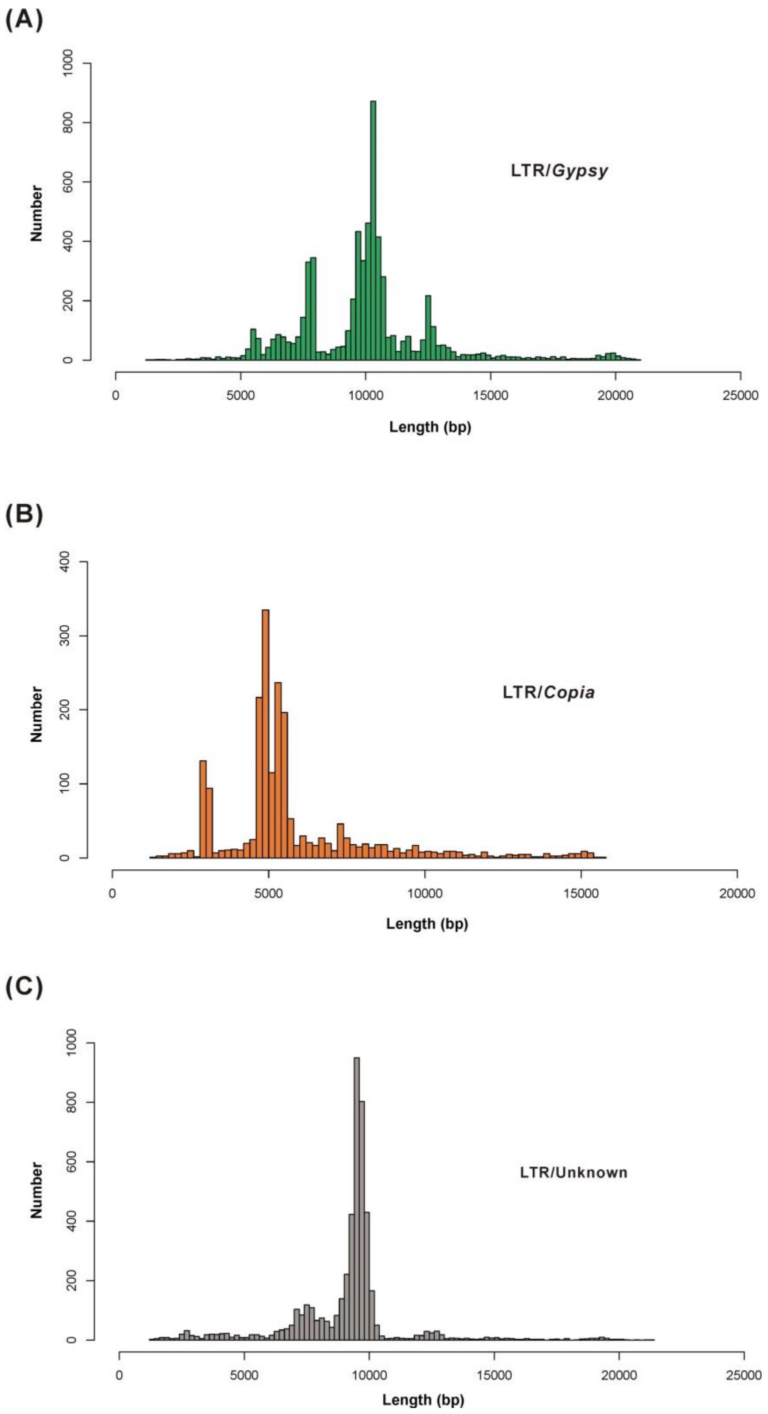

Figure S4. Length distribution of three types of intact LTR-RTs within the *P. uniflora* genome.

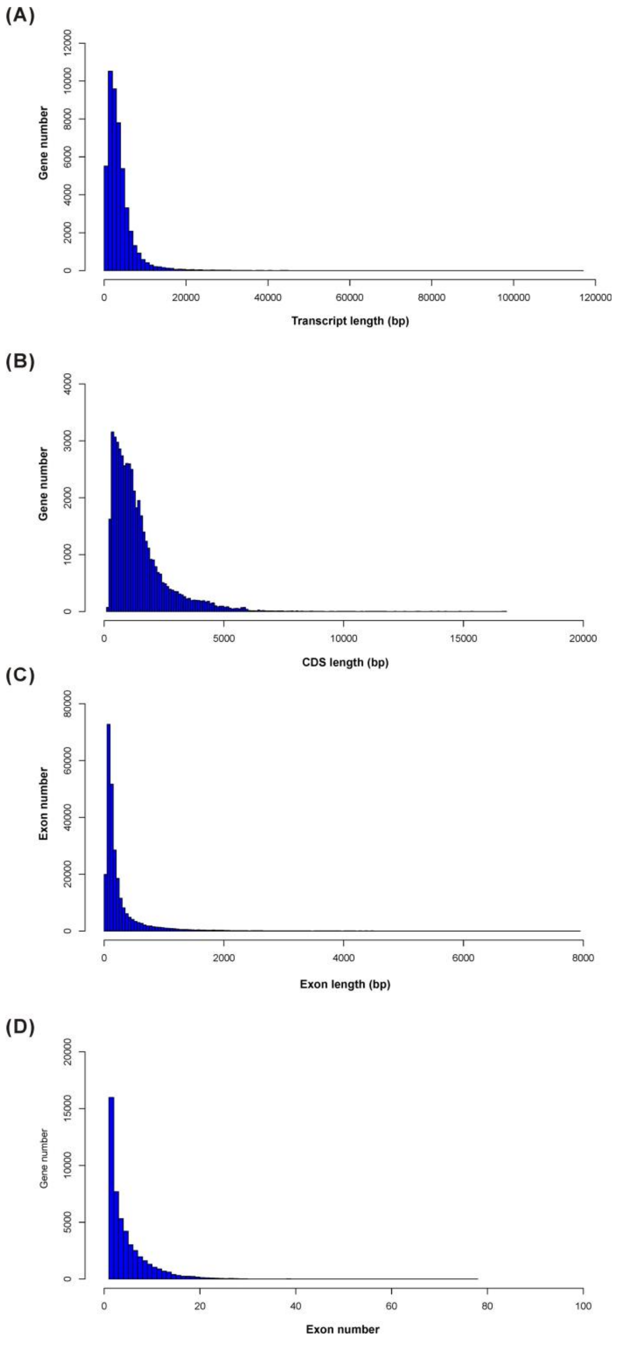

Figure S5. General statistics of protein-coding genes within the *P. uniflora* genome.

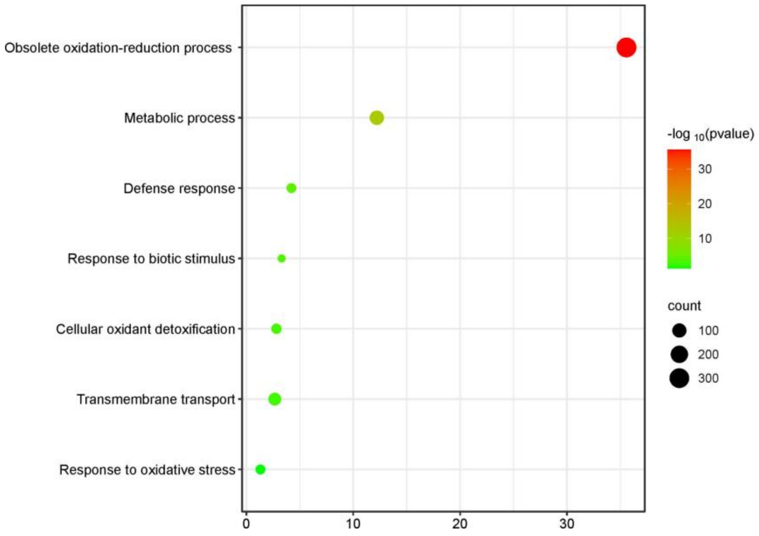

Figure S6. GO enrichment of tandemly duplicated genes within the *P. uniflora* genome.

	Illumina	PacBio HiFi	Hi-C
Number of reads	304,011,762*2	1,639,479	354,952,246*2
Average length of reads (bp)	150	16,328	150
Total bases (Gb)	91.20	26.77	106.49
Sequencing depth (×)*	68.8	20.2	80.3

*The genome size was estimated to be 1,329.36 Mb

Table S1. Summary of the whole genome sequencing data of the *P. uniflora* genome.

	Version 1	Version 2	Version 3
Number of contigs	3,672	865	820
Total length (bp)	1,675,697,640	1,280,423,630	1,272,310,984
GC (%)	41.92	41.76	41.68
Shortest contig (bp)	12,788	12,788	15,609
Longest contig (bp)	16,383,272	16,383,272	16,383,272
Contig N50 (bp)	2,202,736	2,790,945	2,834,015
Contig N90 (bp)	276,877	838,334	856,082

Table S2. Summary of three versions of assemblies of the *P. uniflora* genome, including version 1 (*de novo* assembly of HiFi reads), version 2 (after removing potential duplicate haplotypes), and version 3 (after removing pseudo-contigs).

Superscaffold	Length (bp)	Number of contigs	Number of genes
Chr01	96,324,327	54	3,617
Chr02	93,777,997	58	3,531
Chr03	85,328,045	65	2,924
Chr04	84,812,506	44	3,619
Chr05	83,562,939	58	3,229
Chr06	82,060,278	67	3,170
Chr07	81,142,252	34	2,512
Chr08	79,319,404	46	2,904
Chr09	78,344,120	38	3,631
Chr10	77,664,007	44	3,257
Chr11	77,279,705	55	2,813
Chr12	75,529,320	56	2,742
Chr13	71,387,358	44	2,807
Chr14	68,583,938	48	2,980
Chr15	68,314,794	53	2,684
Chr16	67,276,188	49	2,728
**Total**	**1,270,707,178**	**813**	**49,148**

Table S3. Statistics for the 16 pseudochromosomes of the final *P. uniflora* genome.

	Contig		Scaffold	
	**Size (bp)**	**Number**	**Size (bp)**	**Number**
N90	835,501	462	68,314,794	15
N80	1,289,017	339	71,387,358	13
N70	1,702,879	253	77,279,705	11
N60	2,221,085	189	78,344,120	9
N50	2,774,639	137	79,319,404	8
Longest	16,354,998	-	96,324,327	-
Sequence > 100 kb	1,266,785,236	737	1,271,726,023	19
Sequence > 1000 kb	1,099,424,360	411	1,270,707,178	16
**Total**	**1,272,310,984**	**848**	**1,272,712,484**	**45**

Table S4. Statistics of the final *P. uniflora* genome.

	Assembly		Annotation	
	**Count**	**Ratio (%)**	**Count**	**Ratio (%)**
Complete BUSCOs	1,566	97.03	1,571	97.34
Complete and single-copy BUSCOs	829	51.36	742	45.97
Complete and duplicated BUSCOs	737	45.66	829	51.36
Fragmented BUSCOs	14	0.87	8	0.50
Missing BUSCOs	34	2.11	35	2.17
Total BUSCOs	1,614	100.00	1,614	100.00

Table S5. BUSCO completeness score of assembly and annotation of the *P. uniflora* genome.

Type	Length occupied (bp)	% of genome
DNA	37,247,512	2.93
LINE	4,448,599	0.35
SINE	424	0.00
LTR	735,579,591	57.80
Gypsy	520,616,428	40.91
Copia	219,837,771	17.27
Satellite	2,724,843	0.21
Simple repeat	1,108,226	0.09
Low complexity	205	0.00
Unclassified	113,335,661	8.91
**Total**	**875,996,529**	**68.83**

Table S6. Classification of repetitive elements in the *P. uniflora* genome.

Tissue	Number of reads	Total size (bp)	Accession number
Leaf	47,076,936	6,986,626,476	SRR26058179
Stem	43,112,290	6,387,894,904	SRR26058178
Flower	48,431,662	7,182,882,726	SRR26058176
Root	49,817,502	7,392,646,015	SRR26058177
**Total**	**188,438,390**	**27,950,050,121**	**-**

Table S7. Summary of RNA-seq data from four different *P. uniflora* tissues.

Software	Species	Number of genes	Mean CDS length(bp)	Exons per transcript	Mean exon length(bp)	Mean intron length(bp)
GeMoMa	*Prunus avium*	37,372	1,338	5.6	240	420
	Prunus mume	36,985	1,327	5.5	240	419
	Prunus persica	37,517	1,368	5.7	241	423
	Prunus dulcis	36,798	1,372	5.7	238	423
	Prunus armeniaca	39,131	1,184	5.1	233	428
	Rosa chinensis	38,659	1,355	5.5	245	419
PASA	-	69,894	1,073	7.9	343	426
Glimmerhmm	-	49,442	832	3.8	220	8,051
Augustus	-	123,716	1,250	4.5	276	516
Snap	-	29,477	759	5.5	138	10,113
Genemark	-	44,134	1,108	5.1	216	395
**Final set**		**49,261**	**1,441**	**5.3**	**274**	**557**

Table S8. Statistics of gene models predicted by various approaches.

TF family	Number	TF family	Number	TF family	Number
bHLH	223	TALE	34	CPP	10
MYB	192	ARF	32	NF-YA	10
ERF	169	HSF	32	YABBY	10
NAC	159	GATA	31	CAMTA	9
C2H2	120	AP2	30	RAV	7
WRKY	104	SBP	29	BBR-BPC	6
FAR1	92	NF-YB	22	GeBP	6
bZIP	91	ZF-HD	19	LSD	5
MYB_related	86	ARR-B	18	NF-X1	4
C3H	79	WOX	18	VOZ	4
GRAS	78	BES1	15	Whirly	4
B3	75	NF-YC	15	HB-PHD	3
G2-like	64	DBB	14	HRT-like	3
Trihelix	63	GRF	14	S1Fa-like	2
LBD	62	CO-like	13	SAP	2
MIKC_MADS	54	E2F/DP	13	LFY	1
HD-ZIP	53	Nin-like	13	NZZ/SPL	1
Dof	46	SRS	12	STAT	1
TCP	40	EIL	11		
M-type_MADS	39	HB-other	11		

Table S9. Classification of transcription factor genes in the *P. uniflora* genome.

	Number of genes	Percent (%)
Total	49,261	-
Annotated	45,256	91.87
NR	45,234	91.83
SwissProt	33,116	67.23
KEGG	16,706	33.91
GO	14,696	29.83
eggNOG	42,521	86.32
Unannotated	4,005	8.13

Table S10. Functional annotation of the protein-coding genes in the *P. uniflora* genome.

#Genes/array	#Array	#Gene
2	1,464	2,928
3	263	789
4	107	428
5	47	235
6	23	138
7	14	98
8	9	72
9	7	63
10	5	50
>10	13	326
Total	1,952	5,127
% of all genes	10.41	

Table S11. Summary of tandemly duplicated genes in the *P. uniflora* genome.

**Type**	**Number**	**Average length (bp)**	**Total length (bp)**
miRNA	181	128.08	23,183
tRNA	957	75.52	72,275
rRNA	1,216	271.15	329,717
28S	329	124.82	41,066
18S	117	1,674.85	195,957
5.8S	109	154.14	16,801
5S	661	114.82	75,893
snRNA	726	115.75	84,037
CD-box	468	100.06	46,829
HACA-box	77	125.40	9,656
Splicing	181	152.22	27,552

Table S12. Summary of non-coding RNAs in the *P. uniflora* genome.
